# Protein-free media for cardiac differentiation of hPSCs in 2000 mL suspension culture

**DOI:** 10.1186/s13287-024-03826-w

**Published:** 2024-07-18

**Authors:** Nils Kriedemann, Felix Manstein, Carlos A. Hernandez-Bautista, Kevin Ullmann, Wiebke Triebert, Annika Franke, Mira Mertens, Inês Carvalheira Arnaut Pombeiro Stein, Andreas Leffler, Merlin Witte, Tamari Askurava, Veronika Fricke, Ina Gruh, Birgit Piep, Kathrin Kowalski, Theresia Kraft, Robert Zweigerdt

**Affiliations:** 1 Leibniz Research Laboratories for Biotechnology and Artificial Organs (LEBAO)Department of Cardiothoracic, Transplantation and Vascular Surgery (HTTG)REBIRTH - Research Center for Translational Regenerative Medicine, Hannover Medical School (MHH), Carl Neuberg-Str. 1, 30625 Hannover, Germany; 2 Evotec SE, Hamburg, Germany; 3 Department of Anesthesiology and Intensive Care Medicine, Hannover Medical School (MHH), Hannover, Germany; 4 Institute of Molecular and Cell Physiology, Hannover Medical School (MHH), Hannover, Germany

**Keywords:** hPSC, Cardiomyocytes, Bioreactor, Protein-free differentiation media

## Abstract

**Background:**

Commonly used media for the differentiation of human pluripotent stem cells into cardiomyocytes (hPSC-CMs) contain high concentrations of proteins, in particular albumin, which is prone to quality variations and presents a substantial cost factor, hampering the clinical translation of in vitro-generated cardiomyocytes for heart repair. To overcome these limitations, we have developed chemically defined, entirely protein-free media based on RPMI, supplemented with L-ascorbic acid 2-phosphate (AA-2P) and either the non-ionic surfactant Pluronic F-68 or a specific polyvinyl alcohol (PVA).

**Methods and Results:**

Both media compositions enable the efficient, directed differentiation of embryonic and induced hPSCs, matching the cell yields and cardiomyocyte purity ranging from 85 to 99% achieved with the widely used protein-based CDM3 medium. The protein-free differentiation approach was readily up-scaled to a 2000 mL process scale in a fully controlled stirred tank bioreactor in suspension culture, producing > 1.3 × 10^9^ cardiomyocytes in a single process run. Transcriptome analysis, flow cytometry, electrophysiology, and contractile force measurements revealed that the mass-produced cardiomyocytes differentiated in protein-free medium exhibit the expected ventricular-like properties equivalent to the well-established characteristics of CDM3-control cells.

**Conclusions:**

This study promotes the robustness and upscaling of the cardiomyogenic differentiation process, substantially reduces media costs, and provides an important step toward the clinical translation of hPSC-CMs for heart regeneration.

**Supplementary Information:**

The online version contains supplementary material available at 10.1186/s13287-024-03826-w.

## Background

The application of cardiomyocytes derived from human pluripotent stem cells (hPSC-CMs; either embryonic stem cells: hESCs or induced pluripotent stem cells: hiPSCs) holds enormous potential for regenerative medicine, tissue engineering, in vitro disease modeling, drug development, and other areas [1, 2]. However, this will necessitate the routine production of hPSC-CMs in significant quantities tailored to specific applications.

For example, for the replacement of cardiac tissue lost due to myocardial infarction, it has been estimated that at least one billion in vitro-derived CMs will be required for each patient's heart [3, 4]. Recent proof-of-concept studies have demonstrated successful engraftment of hPSC-CMs in damaged hearts across different animal models and suggested the graft-related improvement of heart function [5–8]. Consequently, these advancements have led to first-in-man studies for hPSC-CM-based heart repair [9, 10]. However, the envisioned routine application of hPSC-CMs to thousands or even millions of heart failure patients in need will require efficient, highly reproducible, and commercially viable cell production processes.

Conventional 2D culture for hPSC expansion and cardiac differentiation relies on complex and costly matrices and hampers process upscaling, monitoring, and control. In contrast, matrix-free 3D suspension culture in stirred culture platforms such as spinner flasks and, in particular, fully controlled stirred tank bioreactors (STBRs) overcomes such limitations. The suspension culture approach has successfully been applied to both hPSC expansion [11, 12] and directed differentiation, including mesodermal derivatives such as cardiomyocytes [13–18] and macrophages [19, 20], endothelial [21], and endodermal derivatives [22].

Culture media are pivotal in process development, impacting process robustness, costs, and regulatory compliance. Commonly used media for the cardiac differentiation of hPSCs, such as RPMI/B27 (consisting of 19–22 components depending on the formulation, including bovine serum albumin, human recombinant insulin, catalase, superoxide dismutase, transferrin, and glutathione reductase) [23], or chemically defined medium, 3 components (CDM3, consisting of only three components, including recombinant human albumin) [24], however, contain high levels of either bovine serum albumin (BSA), or recombinant human serum albumin (rHSA), respectively. Albumin is known to regulate the osmotic pressure, bind and stabilize lipids, proteins, and metal ions, and possess antioxidant functions, both in vivo and in vitro [25]. Furthermore, albumin has been suggested to have a shear-protectant effect, thereby promoting cell viability in stirred and especially gas-sparged processes [26].

However, albumin and other proteins are susceptible to batch-dependent quality variations, represent a substantial cost factor, and notably reduce the sensitivity of proteome/secretome analysis in media samples [27–29], thus hampering the understanding of complex differentiation processes and in-process-control analytics of secreted factors [30, 31].

This in mind, we here investigated the replacement of albumin, enabling the entirely protein-free production of hPSC-CMs in suspension culture. We found that RPMI medium, supplemented with the ascorbic acid derivative AA-2P combined with either the poloxamer Pluronic F-68 (PF-68) or a specific polyvinyl alcohol (PVA; ⌀85,000–124000 Da; hydrolyzation of 87–89%) promotes the production of hPSC-CMs equivalent to protein-containing control conditions using CDM3. The resulting differentiation media are completely chemically defined, only containing components which, in contrast to proteins, are not prone to quality alterations and, in addition, reduce the overall media costs [32].

Following formulation development in 20 mL scale in shaker flasks, systematic upscaling of the process to 150 mL and, ultimately, a 2000 mL stirred-tank bioreactor scale was performed. Through this approach, we demonstrated the production of > 1.3 × 10^9^ CMs per 2000 mL batch at purities exceeding 85% hPSC-CM content while maintaining cells' properties equivalent to CDM3 controls. For downstream applications, such as cell therapies to the heart, the generated CM-aggregates may be directly amenable for transplantation, presenting a viable alternative therapy strategy compared to single CMs [8, 33].

## Methods

### hPSC lines and adherent monolayer culture

A human embryonic stem cell (hESC) line containing a mix paired-like homeobox transcription factor 1 (MIXL1)-GFP reporter gene [34], termed HES3 MIXL1-GFP (female donor), was used for process development in Erlenmeyer flasks and STBRs. The reporter gene enables an early and direct readout of differentiation toward the primitive streak. This cell line was obtained from E. G. Stanley and A. G. Elefanty (Monash Immunology and Stem Cell Laboratories, Monash University). The hiPSC line Phoenix (HSC_ADCF_SeV-iPS2; MHHi001-A; female donor) from CD34 + human cord blood hematopoietic cells was, in addition, used for the demonstration of the general applicability of the developed differentiation media [35]. In addition, we used the cell line hHSC_1285i_iPS2 (MHHi006-A; female donor) for the upscaling into the 2000 mL bioreactor [36]. Furthermore, an hiPSC cell line generated under GMP-like conditions from CD34^+^ hematopoietic stem cells reprogrammed by transduction with a Sendai virus vector termed GMPDU_8 or GMPDU for short was utilized (CD34 + hPBHSC_GMPDU_SeV-iPS8; MHHi008-A; male donor) [37]. All hPSC lines were precultured in adherent 2D monolayer culture on Geltrex (Thermo Fisher Scientific)-coated T-flasks (Greiner) in E8 medium (supplemented with 10 µm Y-27632) [38]. After 48 h, the medium was exchanged for fresh E8 medium. After 72 h, the hPSCs were detached with Accutase (Thermo Fisher Scientific) for 3 min at 37 °C and transferred to new Geltrex-coated T-flasks or into suspension culture [39]. All hPSC lines were passaged three times before inoculation of suspension culture.

### Differentiation media composition

Multiple media compositions were investigated in this study. All were based on an RPMI 1640 medium basis, supplemented with 5958 mg/L 4-(2-hydroxyethyl)-1-piperazineethanesulfonic acid (HEPES) and 300 mg/L L-Glutamine (Cat. No. 22400089; Thermo Fisher Scientific). Furthermore, L-ascorbic acid 2-phosphate (AA-2P) at 213 µg/mL was added to all media (Merck). Medium containing only RPMI 1640 and AA-2P was termed Basis (BA) medium. Furthermore, the medium was supplemented with Pluronic F-68 (Thermo Fisher Scientific) at a final concentration of 0.1% (BA + PF-68). Moreover, we investigated the use of low concentrations of transferrin (final transferrin concentration 10.7 µg/mL; Merck) (BA + Transferrin). Also, a range of polyvinyl alcohols (termed PVA1, PVA2, PVA3) was investigated. PVA1 had an average molecular weight of 85,000–124,000 Da and a hydrolyzation grade of 87–89% (Merck, Cat. No. 363081; BA + PVA1). PVA2 had a hydrolyzation grade of 87–90% but a lower average molecular weight of 30,000–70,000 Da (Merck; Cat. No. P8136; BA + PVA2). PVA3 had a higher hydrolyzation of 99 + % and an average molecular weight of 85,000–124,000 Da (Merck; Cat. No. 363146; BA + PVA3). All PVAs were used at a final concentration of 0.1%.

### Bioreactor set-up

hPSCs were expanded and differentiated in an Eppendorf Mini Bioreactor system DASbox and Bioblock (Eppendorf). The glass bioreactors (flat-bottom) were equipped with an eight-blade pitched impeller, a supply port for base to regulate the pH, gas supply via the headspace, a supply port for fresh medium, and a port with a 20–40 mm porous glass filter for removal of the used medium. Further, a sampling port for aseptic sample taking was equipped with a needleless injection Luerlock port. Peristaltic pumps (Eppendorf) supplied fresh medium, removed used medium, or supplied base according to settings in the bioreactor software.

Further, the bioreactors were equipped with pH sensors (Mettler Toledo) and sensors for dissolved oxygen (DO) (Hamilton Company). All sensors and pumps were calibrated according to the manufacturer's protocols. A detailed protocol for the bioreactor set-up was published before [39]. The 2000 mL system (Bioflo 320, Eppendorf) had a hemispherical bottom, in contrast to the DASbox bioreactor, and was instead equipped with a three-blade impeller, but otherwise, it had the same technical features.

### hPSC expansion and cardiac differentiation in suspension culture

hPSCs were inoculated as single cells in E8 medium at a cell density of 0.5 × 10^6^ viable cells/mL. Further, Rho-associated kinase inhibitor Y-27632 (RI) was added to the medium at a concentration of 10 µM to prevent anoikis, and PF-68 was added at a concentration of 0.1% [11]. The hPSCs form cell-only aggregates and are cultivated for two to three days (depending on the cell line) in suspension at 80 rpm in STBRs. After 24 h of cultivation, perfusion starts in STBRs, exchanging 1 × reactor volume/day for fresh medium with an increased glucose concentration of 6 g/L (versus 3 g/L glucose under standard conditions). After 48 h of hPSC expansion, the medium exchange rate was automatically adjusted to 1.5 × reactor volumes/day, as described before [11]. On days two/three, aggregates were sampled, and the viable cell density was determined after dissociation of aggregates with Accutase and automated cell counting with a ViCell XR device (Beckmann Coulter). Then, 75 × 10^6^ viable hPSCs as aggregates were collected through centrifugation, resuspended in differentiation medium supplemented with 5 µM CHIR (Institute for Organic Chemistry, Leibniz University Hannover and Tocris Bioscience), and reinoculated in a bioreactor at 150 mL scale. Accordingly, for a 2000 mL bioreactor scale, 1 × 10^9^ viable hPSCs were inoculated. After precisely 24 h, the medium was exchanged entirely for the respective medium supplemented with 5 µM IWP-2 (Tocris Bioscience). After 72 h, the complete medium was exchanged for the respective differentiation medium without adding small molecules. From 96 h process time onwards, the medium was exchanged at 1/3 reactor volume per day via perfusion. Differentiated aggregates were analyzed on day 10 of differentiation for the presence of cardiac markers. The differentiation was performed at a 150 mL scale at 70 rpm in STBRs or 68 rpm in the 2000 mL system.

In Erlenmeyer flasks, 10 × 10^6^ viable hPSCs as aggregates were inoculated in the respective differentiation medium supplemented with 5 µM CHIR. After 24 h, the medium was exchanged for the respective differentiation medium supplemented with 5 µM IWP-2. After 72 h, the medium was exchanged entirely for plain differentiation medium and, afterward, exchanged every two days. Also, the CM yield and purity were analyzed on day 10 of differentiation.

The general hPSC expansion strategy and differentiation protocol, as well as the method to determine aggregate size were published previously [11, 13].

### Cardiomyocyte aggregate dissociation for downstream analysis

A cell sample was taken from the suspension culture and dissociated to obtain single cells to analyze the cell population throughout the process and at the endpoint. When performed with aggregates during pre-culture or on the first three days of the differentiation process, dissociation was performed with Accutase (Thermo Fisher Scientific) for 3 min in an Eppendorf Thermomixer at 37 °C. After day 3 of differentiation, the STEMcell technologies cardiomyocyte dissociation kit was utilized for 3–6 min in an Eppendorf Thermomixer at 37 °C, with increasing incubation time towards the end of the process. The dissociation was stopped by diluting the dissociation reagent with cardiomyocyte support medium at a 3:1 ratio. After centrifugation and resuspending, cells were available for counting, flow cytometry analysis, or seeding for electrophysiological or immunohistochemical analysis or the production of BCTs.

To seed single CMs for patch clamp analysis and immunofluorescence staining, cells were kept in their differentiation medium until day 17 of differentiation and dissociated as described above. After dissociation, single cells were resuspended in medium consisting of IMDM + GlutaMAX (Thermo Fisher Scientific), supplemented with 20% FCS (Cytiva), 1 mM L-glutamine (Thermo Fisher Scientific), 1% non-essential amino acids (Thermo Fisher Scientific), 0.1 mM 2-mercaptoethanol (Thermo Fisher Scientific), 10 µM Y-27632 (Tocris Bioscience) and 1% penicillin/streptomycin (Merck). 10,000 cells were applied to glass slides coated with 0.1% gelatin (Merck) and 4 µg/mL fibronectin (Corning) [13]. One day after seeding, the medium was exchanged for RPMI + B27 medium. The medium was renewed every 2 days, and cells were further analyzed after 7 days.

### Electrophysiological analysis

Seeded hPSC-CMs were analyzed in whole-cell mode with an EPC10 amplifier using the Patchmaster v20 × 60 software (HEKA instruments). Data was analyzed using Fitmaster software (HEKA instruments). Signals were filtered at 5 kHz and sampled at 20 kHz. Pipettes (GB150EFT-10, Science Products) were pulled on a DMZ-Universal Puller (Zeitz) and heat polished to give a resistance of ~ 2 MΩ when filled with pipette solution. For current-clamp recordings of APs, the extracellular solution contained 140 mM NaCl, 4 mM KCl, 2 mM CaCl_2_, 1 mM MgCl_2_, 10 mM HEPES, and 10 mM glucose. The pipette solution contained 140 mM KCl, 4 mM MgCl_2_, 10 mM HEPES, and 10 mM EGTA. Both solutions were adjusted to pH 7.4 with NaOH. The offset potential was zeroed before the cells were patched; the junction potential was not corrected for. Patch clamp data was analyzed using Pulsefit software (HEKA Instruments Inc.) Data analyses were performed with Origin 6.0 (Microcal Software).

### Immunofluorescence staining of hPSC-CMs

Cells were fixed with 4% PFA at room temperature for 10 min for immunofluorescence staining. Then, cells were washed with PBS w/o and blocked with 5% (w/v) donkey serum (Merck), 0.25% (v/v) Triton X-100 (Merck) in Tris-buffered saline (TBS) at RT for 20 min. Cells were incubated with the primary antibody diluted in TBS (containing 1% w/v BSA; Merck) at 4 °C overnight. Afterward, cells were washed with TBS, and the secondary antibody was diluted in TBS and added. After a 30-min incubation at RT, cells were washed again with TBS. To stain nuclei with DAPI, 1.7 µg/mL in PBS + Ca^2+^/Mg^2+^ was added to the cells and incubated for 15 min at room temperature. The stained cells were then analyzed with an Axio Observer A1 fluorescence microscope and Axiovison software (Zeiss).

### Confocal microscopy of hPSC-CM aggregates

Aggregates were prepared for confocal microscopy as recently described [18]. In short, aggregates were stained with Sytox Deep Red, dehydrated with EtOH, and cleared with methyl salicylate/benzyl benzoate. With a confocal microscope (e.g., Zeiss LSM 980 Airyscan), internal structures in intact aggregates were revealed.

### Production of bioartificial cardiac tissues

Bioartificial cardiac tissues (BCTs) were produced from differentiated cardiac aggregates after dissociation into single cells, as described previously [40]. The cell–matrix solution was prepared with 1.34 mg/mL rat tail collagen type I (Thermo Fisher), 0.014 M NaOH, and 0.012–0.017 mg/mL Geltrex™ (Gibco), together with 1 × 10^6^ cardiomyocytes and 0.1 × 10^6^ irradiated human foreskin fibroblasts (irr-hFF) per BCT in BCT medium (DMEM supplemented with 12% Horse Serum (Gibco), 1% L-glutamine, 1% penicillin–streptomycin). The cell–matrix mixture was poured into custom-made silicon molds lined with two titanium rods placed 6 mm apart (initial slack length). After solidification for 30 min at 37 °C, 5 mL BCT medium, supplemented with 60 μM L-ascorbic acid, was added per tissue, and medium renewal was performed every second day. Growing static stretch, increasing in 400 μm increments, was applied every fourth day between days 7 and 19. 21 days after production, the BCTs were transferred to culture vessels to perform force measurements using a multimodal bioreactor [41].

### Flow cytometry

Day 1 differentiating aggregates (for MIXL1-GFP expression analysis) or day 10 cardiac aggregates were harvested for flow cytometry analysis. Gathered aggregates were washed with PBS and dissociated, either with Accutase (Thermo Fisher Scientific) for day 1 differentiating aggregates or with STEMdiff cardiomyocyte dissociation kit (Stemcell Technologies) for CM aggregates on day 10 as described before. After washing with PBS, day 1 single cells derived from differentiating aggregates were directly analyzed for their expression of MIXL1-GFP via flow cytometry. Day 10 cardiac aggregates were treated with a Fixation and Permeabilisation Flow Cytometry Kit (Novus Biologicals) and stained with the respective primary antibody for 1 h at room temperature. After washing, the cells were incubated for 30 min with a respective APC-conjugated secondary antibody. After further washing, the cells were analyzed with a MACSQuant flow cytometer (Miltenyi Biotec). Utilized antibodies can be found in Additional File 1, Table S1.

### RNA isolation and Bulk RNA sequencing

CM aggregates equivalent to 3 × 10^6^ viable cells were derived from the process, centrifuged, and resuspended in 500 µL TRIzol reagent (Thermo Fisher Scientific). Aggregates were mechanically disrupted by vortexing until the solution was free from visible cells. RNA samples were stored at -80 °C until further processing. After thawing, 100 µL of chloroform (Merck) was added and centrifuged at 12,000 × *g* at 4 °C for 15 min. The aqueous phase containing the cellular RNA was separated and processed using a NucleoSpin RNA Kit II (Machery-Nargel). The RNA quantity and purity were measured via UV-spectrophotometry. 500 ng of total RNA was used as input for mRNA enrichment with NEBNext Poly(A) Magnetic Isolation Module (New England Biolabs). A stranded cDNA library was generated using the NEBNext Ultra II Directional RNA Library Prep Kit for Illumina (New England Biolabs). The library pool was denatured with NaOH and diluted to 1.8 pM. 1.3 mL of denatured library pool was loaded on an Illumina NextSeq 550 sequencer using a High Output Flowcell. The resulting BCL files were converted to FASTQ files using bcl2fastq Conversion Software (Illumina), and the data was processed with nforce/rnaseq (version 3.9; 10.5281/zenodo.1400710). Normalization and differential expression analysis were performed on the internal Galaxy instance of the Hannover Medical School RCU Genomics.

### Western blot analysis for cytoskeletal proteins and channels

Aggregates were collected on dd16/17 and frozen in liquid nitrogen. Cells were lysed in kinase buffer (20 mM Tris–acetate, pH 7.0, 0.1 mM EDTA, 1 mM EGTA, 1 mM Na3VO4, 10 mM β-glycerolphosphate, 50 mM NaF, 5 mM pyrophosphate, 1% Triton X-100, 2 µg/mL Leupeptin; 0.27 M sucrose, supplemented with Protease Inhibitor Cocktail (Roche) and PhosSTOP Phosphatase Inhibitor Cocktail (Roche)) [42] to analyze the abundance of various cytoskeletal proteins and typical channels expected to be present in CMs. The cell lysate was mixed (4:1) with ROTILoad1 (Roth) and heated for 4 min to 85 °C and then separated on an SDS Page gel (BioRad Criterion Gel 4–15% or 7.5%) and transferred via Criterion blotter (Biorad). The blotted proteins were stained with the respective antibody or SYPRO Ruby (Biorad) for α-/β-Myosin, and the intensity (optical density) of the respective band was quantified using Imagequant software V8.2 (Cytiva) and the LAS400 system (Cytiva). All samples were normalized to either a standard or α-actinin to account for differences in the applied amount of protein and compared to one CDM3 sample to allow cross-gel comparison. Samples from the human heart were used as a control for the position of α/β-Myosin on the SDS gels. Utilized antibodies can be found in Additional File 1, Table S2.

### Statistical analysis

All experiments were performed at least in three independent biological replicates as long as not stated differently. Data was analyzed with GraphPad Prism 8 (GraphPad Software Inc.) unless otherwise indicated. Data is presented as mean ± standard deviation (s.d.) or standard error of the mean (SEM), as noted in the respective figure.

## Results

### Supplementation with Pluronic F-68 or a specific polyvinyl alcohol allows for the efficient protein-free cardiac differentiation of hPSCs in suspension culture

**Fig. 1:**
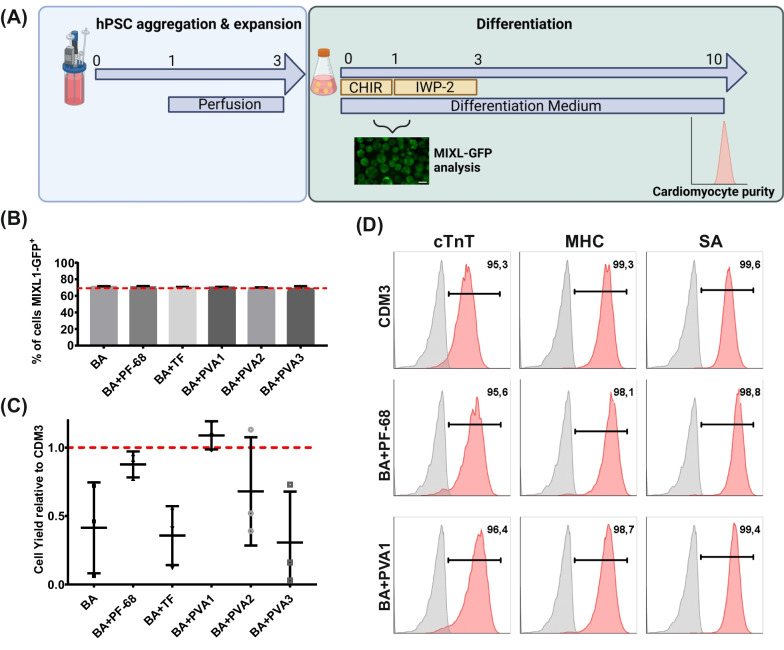
BA + PF-68 and BA + PVA1 achieve CM yield and purities comparable to CDM3 in shaken Erlenmeyer flasks. **A** Schematic of differentiation. hPSC aggregates are formed over three days of expansion in STBRs and transferred to Erlenemeyer flasks, where different differentiation media were used. **B** Expression of MIXL1-GFP on dd1 for the investigated differentiation media assessed by flow cytometry compared to CDM3 (dashed line; n = 3; results are mean ± s.d.). **C** Cell yields on dd10 for respective media formulations compared to CDM3 cell yields (dashed line; n = 3; results are mean ± s.d.). **D** Representative flow cytometry plots revealing the expression of investigated CM markers cardiac troponin T (cTnT), pan-myosin heavy chain (MHC), and sarcomeric actinin (SA) for CDM3, BA + PF-68, and BA + PVA1

The schematic in Fig. 1A depicts our strategy for investigating how different protein-containing or protein-free media (outlined in Table 1) support the cardiac differentiation of hPSCs in suspension culture in shaken Erlenmeyer flasks. To ensure uniform culture conditions, hPSCs were expanded in conventional monolayer culture for three passages for stirred tank bioreactor (STBR) inoculation with single hPSCs at a density of 0.5 × 10^6^/mL for cell-only aggregate formation according to recent work [11]. After three days, the viable cell number was determined, and aggregates equivalent to about 10 × 10^6^ hPSCs were transferred to 20 mL of differentiation medium supplemented with 5 µM CHIR (for chemical WNT pathway induction) into Erlenmeyer flasks shaken at 70 rpm [13]. The concentration of respective media supplements was chosen based on published optimization studies that are: 0.1% v/v for Pluronic F-68 (PF-68) [11], 10.7 mg/L w/v for transferrin [38], and 0.1% v/v for polyvinyl alcohols (PVAs) [43]. Exactly 24 h after CHIR-based induction of differentiation, the medium was replaced for the same respective differentiation medium but supplemented with 5 µM IWP-2 (for chemical WNT pathway attenuation). This medium was replaced after 48 h for differentiation medium without small molecules and refreshed every 2 days (Fig. 1A).

**Table 1:** Composition and designation of investigated cardiomyocyte differentiation media

	Basis (BA)	CDM3	BA + PF-68	BA + TF	BA + PVA1	BA + PVA2	BA + PVA3
RPMI-1640	X	X	X	X	X	X	X
L-ascorbic acid 2-phosphate (AA-2P Vitamin C Derivative); 213 mg/L	X	X	X	X	X	X	X
Recombinant human albumin (RHA); 500 mg/L		X					
Pluronic F-68 (PF-68); 0.1%			X				
Transferrin (TF); 10.7 mg/L				X			
Polyvinyl alc. 1 (PVA1) ⌀ 85,000–124,000 Da; hydrolyzation 87–89%; 0.1%					X		
Polyvinyl alc. 2 (PVA2) ⌀ 30,000–70,000 Da; hydrolyzation of 87–90%; 0.1%						X	
Polyvinyl alc. 3 (PVA3) ⌀ 85,000–124,000 Da; hydrolyzation of 99 + %; 0.1%							X

Using a MIXL1-GFP-reporter hESC line, primitive streak-like priming was monitored by flow cytometry on day 1 (dd1) of differentiation [30, 34, 44–46] (Fig. 1B). A highly similar transgene expression of approximately 70% was observed across all media tested (reflecting the proportion of positive cells previously described in RPMI + B27 without insulin [30]), indicating that albumin is not required for small molecule (i.e., CHIR)-driven induction of mesendodermal differentiation [30]. On dd10, the viable cell yield and CM content were investigated. In contrast to the heterogenous results observed for “Basis” (BA) medium as well as BA + PVA2, BA + PVA3, and BA + TF formulations (Fig. 1C), the two compositions BA + PVA1 and BA + PF-68 promoted highly reproducible cell yields and CM purities, closely reflecting the benchmark differentiation results in CDM3 **(**Fig. 1C; Fig. 1D). Based on these small-scale process development studies in the Erlenmeyer flask platform, BA + PVA1 and BA + PF-68 where further tested for differentiation process upscaling in STBR-based experiments.

### Protein-free conditions promote cardiac differentiation in STBRs

Applying a 150 mL process scale in an impeller-stirred STBR platform, the formulations BA + PVA1 and BA + PF-68 were tested applying a differentiation protocol previously established with CDM3, typically resulting in 1 × 10^6^ hPSC-CMs/mL, yielding 150 × 10^6^ hPSC-CMs per process batch in 10 days [13].

**Fig. 2:**
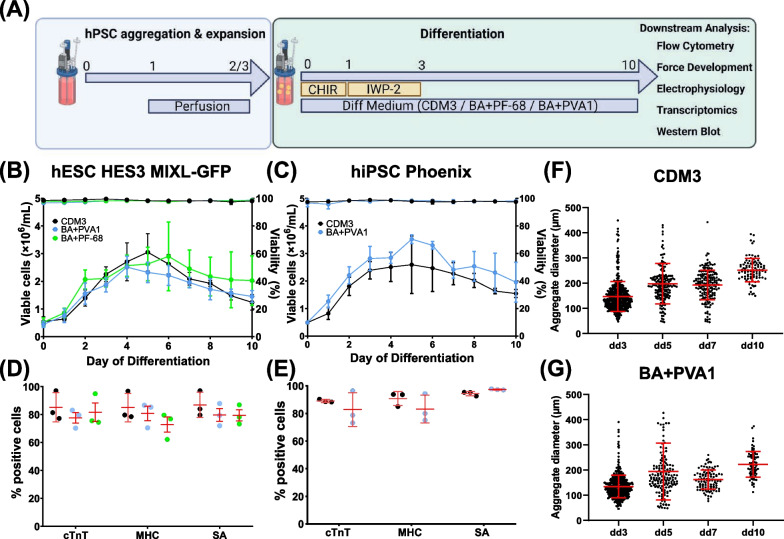
Protein-free differentiation media closely resemble typical patterns observed for CDM3 at 150 mL process scale.** A** Schematic of differentiation. hPSC aggregates were formed over two or three days (depending on cell line). The viable cell density was adjusted to 75 × 10^6^ hPSCs per 150 mL bioreactor, and the differentiation was performed. **B** Viable cell number and viability for the hESC line HES3 MIXL1-GFP for CDM3 (black), BA + PVA1 (blue), BA + PF-68 (green) throughout the 10-day lasting differentiation process (n = 3 for each medium; mean ± s.d.) and **C** for the hiPSC line Phoenix HSC_ADCF_SeV_iPS2 for CDM3 (black) and BA + PVA1 (blue; n = 3 for each medium; mean ± s.d.). **D** CM-specific markers cTnT, pan-MHC, and SA for the hESC line MIXL1-GFP for CDM3 (black), BA + PVA1 (blue), BA + PF-68 (green; n = 3 for each medium; mean ± s.d.) and **E** for the hiPSC line Phoenix for CDM3 (black) and BA + PVA1 (blue; n = 3 for each medium; mean ± s.d.). **F** Aggregate size development during differentiation for exemplary processes in CDM3 or **G** BA + PVA1 (cell line Phoenix; each dot represents a single analyzed aggregate, red lines indicate mean ± s.d.)

Before differentiation, hPSCs were expanded at yield-optimized suspension conditions in E8 medium, exemplarily depicted in Additional File 1, Figure S1 [11]. To ensure the general cell line-independent applicability of our approach (schematically outlined in Fig. 2A), growth and differentiation characteristics with protein-free formulations were investigated with the lines hESC MIXL1-GFP and hiPSC Phoenix (focus on BA + PVA1) and compared to the established conditions in CDM3 [13].

At the induction of differentiation at dd0, hPSC aggregates were in a size range of 100–200 µm (Additional file 1, Figure S1B + C), favorable for cardiac differentiation [18, 30, 47, 48]. At the start of differentiation, the cell density was adjusted to 75 × 10^6^ in 150 mL medium, and differentiation was induced by CHIR [13]. Viable cell numbers constantly increased up to dd4-6, followed by a steady decline as previously observed [13]; growth kinetics of protein-free media closely resembled the pattern for CDM3. Cell counts on dd10 were as follows for i) hESC MIXL1-GFP; CDM3: 1.24 ± 0.28 × 10^6^; BA + PVA1: 1.45 ± 0.22 × 10^6^; BA + PF-68: 2.02 ± 0.9 × 10^6^ and ii) for hiPSC Phoenix: CDM3: 1.54 ± 0.15 × 10^6^; BA + PVA1: 1.96 ± 0.72 × 10^6^ viable cells/mL (Fig. 2B + C).

Importantly, according to the CM-specific markers cTnT, pan-MHC, and SA, the CM content in protein-free media was also highly similar to CDM3 for the above-noted lines hESC MIXL1-GFP and hiPSC Phoenix (Fig. 2D + E) and further confirmed for two additional, independent hiPSC lines in 150 mL STBR scale (Additional File 1, Figure S2). Aggregate size patterns were also highly similar as exemplified for BA + PVA1 compared to CDM3 (Fig. 2F + G). As the overall results for both protein-free media were comparable, we further focused on the in-depth analysis of BA + PVA1-derived CMs.

### CMs derived under protein-free conditions are highly similar to CDM3-based controls regarding their molecular and physiological properties

**Fig. 3:**
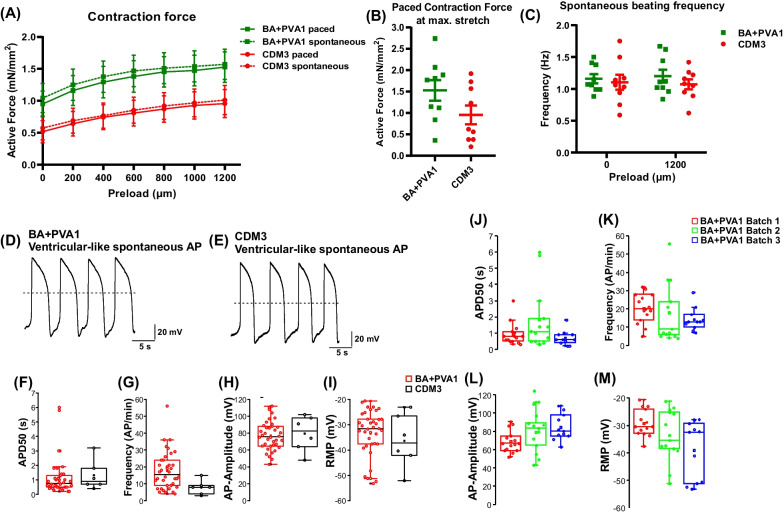
Contraction and electrophysiological analysis revealed no substantial differences between CMs from protein-free differentiation and CDM3. **A** Bioartificial cardiac tissues (BCTs) were produced from three independent differentiation runs in either BA + PVA1 or CDM3 medium (cell line Phoenix, 3 BCTs per biological replicate for 3 biological replicates; mean ± SEM). The paced and spontaneous contraction forces increase to a similar level (mN/mm^2^) with increasing preload. The values were normalized to the diameter of the individual BCT. **B** Individual BCT forces (mN/mm.^2^) at the maximum stretch (1200 µm; mean ± SEM). **C** Spontaneous beating frequency (Hz) for BCTs from CMs differentiated in BA + PVA1 or CDM3 at 0 µm and 1200 µm preload (mean ± SEM). **D** Representative ventricular-like action potentials (APs) for BA + PVA1-derived and **E** CDM3-derived, seeded CMs were revealed by patch clamp analysis. **F** Duration of the action potential at 50% of the amplitude (APD50) for CMs derived from BA + PVA1 and CDM3 conditions. **G** Frequency of APs/min for CMs derived from BA + PVA1 and CDM3 conditions **H** AP amplitude (in mV) for CMs derived from BA + PVA1 and CDM3 conditions. **I** Resting membrane potential (in mV) for CMs derived from BA + PVA1 and CDM3 conditions (for BA + PVA1 n = 3 biological replicates, for CDM3 control n = 1 in F-I) **J-M** Comparison of APD50, AP frequency, AP amplitude, and RMP in three biologically independent CM batches produced in BA + PVA1 (all mean ± SEM)

For more detailed investigations on media-dependent CM characteristics, contractile forces were assessed via bioartificial cardiac tissues (BCTs) [13, 49, 50]. BCTs produced from BA + PVA1-differentiated CMs exerted equivalent or, by tendency, higher (i) spontaneous active and (ii) electrically paced active contraction forces compared to CDM3 controls under increasing preload, as well as (iii) at the endpoint at the maximum stretch (Fig. 3A + B). Respective BCTs were equivalent in spontaneous beating frequency and closely resembled previous results observed from protein-dependent differentiations utilizing the same cell line (Fig. 3C) [13].

Single-cell patch clamp analysis of suspension-derived CMs seeded in 2D revealed ventricular-like CMs according to their plateau phase lasting > 200 ms at 50% of their repolarization level (APD50) [17, 53, 55, 56]. Indeed, under protein-free and CDM3 conditions, the majority of CMs showed the typical action potential shape of immature, ventricular-like CMs, as depicted in Fig. 3D + E. Under both conditions, the measured APD 50 was between 0.5 and 1 s (Fig. 3F). Moreover, the AP frequency, AP amplitude, and resting membrane potential (RMP) were highly similar (Fig. 3G–I). When comparing patched CMs derived from independent differentiation batches from BA + PVA1, highly reproducible values for APD50, AP frequency, AP amplitude, and RMP were observed (Fig. 3J–M).

As expected, aggregate-derived CMs seeded in monolayer showed typical sarcomeric structures after immunofluorescence stainings (Additional File 1, Figure S3A-D) [18]. Confocal microscopy revealed a similar 3D cardiac aggregate morphology in BA + PVA1 and CDM3 conditions (Additional File 1, Figure S3E + F). The amount of proteins for various CM-typical channels (Nav1.5, RyR2, NCX, Cav1.2, and Kir2.1) and structural proteins (α/β-myosin, MYLC1a/v, TPM-α/β), including the predominant expression of α-myosin was also equivalent. The dominant tropomyosin isoform was TPM-α, and mainly MYLC1v was expressed. For none of the investigated proteins, significant media-dependent differences were found (Additional File 1, Figure S4).

**Fig. 4:**
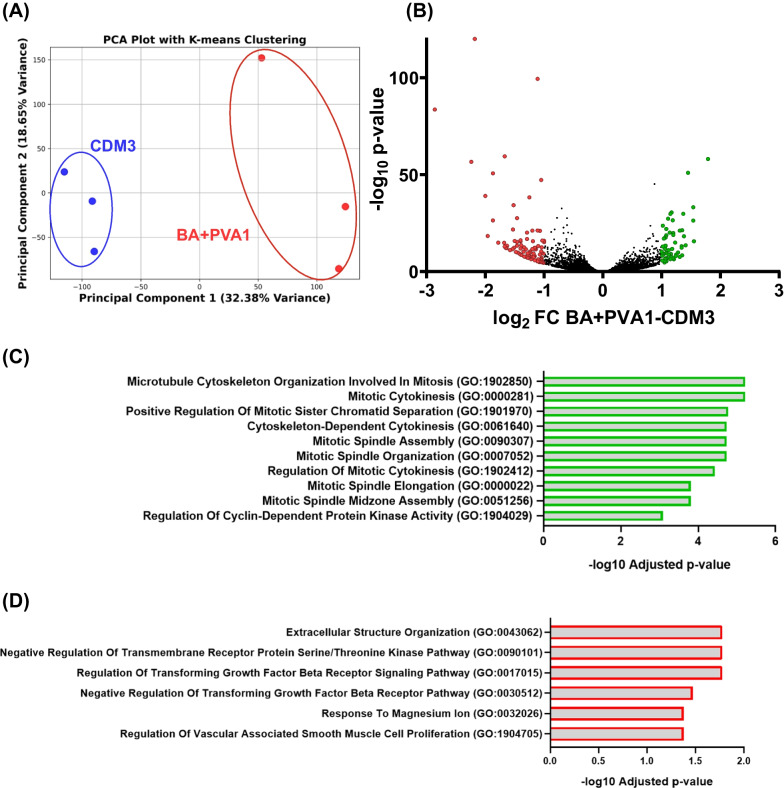
Transcriptomic analysis of CMs from BA + PVA1 and CDM3 revealed significant differences. **A** Principal component analysis of three biologically independent replicates of CMs from BA + PVA1 (red) and CDM3 (blue) conditions according to K-means clustering. **B** Volcano plot of transcripts identified in all samples with ≥ 10 counts. Significantly up/downregulated transcripts (*p* ≥ 0.05) with a fold change ≥ 1/ ≤ − 1 between the two groups are marked in green or red, respectively. Three independent biological samples with comparable cTnT purity (according to flow cytometry analysis) were analyzed for both conditions. **C + D** Gene Ontology analysis of the most significant results between CMs differentiated in BA + PVA1 or CDM3 to be upregulated in BA + PVA1 (**C**) or downregulated in BA + PVA1 (**D**). Only genes of transcripts with a fold change ≥ 1/ ≤ − 1 and *p* ≥ 0.05 were considered

BA + PVA1 versus CDM3-derived CM batches (all at comparable CM purities, n = 3 for each condition) were also compared by bulk-RNA-sequencing. According to principal component analysis, respective samples differentiated in BA + PVA1 or CDM3 clustered together, suggesting some degree of media-dependent expression patterns (Fig. 4A). Among the 16,185 identified transcripts (count > 10 in each sample), 61 were significantly upregulated with a log_2_ fold-change > 1 in BA + PVA1-derived CMs compared to CDM3 CM samples. 81 transcripts were significantly downregulated (Fig. 4B; Additional File 1, Table S3). However, we did not observe significant, biologically relevant (log_2_ fold-changes > 1) changes in the expression of typical CM-specific genes such as TNNT2, NKX2.5, ACTC1, TNNI1, etc. Interestingly, Gene Ontology (GO) analysis [52] of transcripts with the highest fold changes indicated increased expression of proliferation-related GOs under protein-free conditions. On the other hand, downregulation of extracellular matrix organization-associated GOs as well as TGF-β signaling, was suggested in BA + PVA1-differentiated CMs (Fig. 4C + D).

### Successful upscaling of CM production from 150 to 2000 mL is compatible with the maintenance of lineage purity

**Fig. 5:**
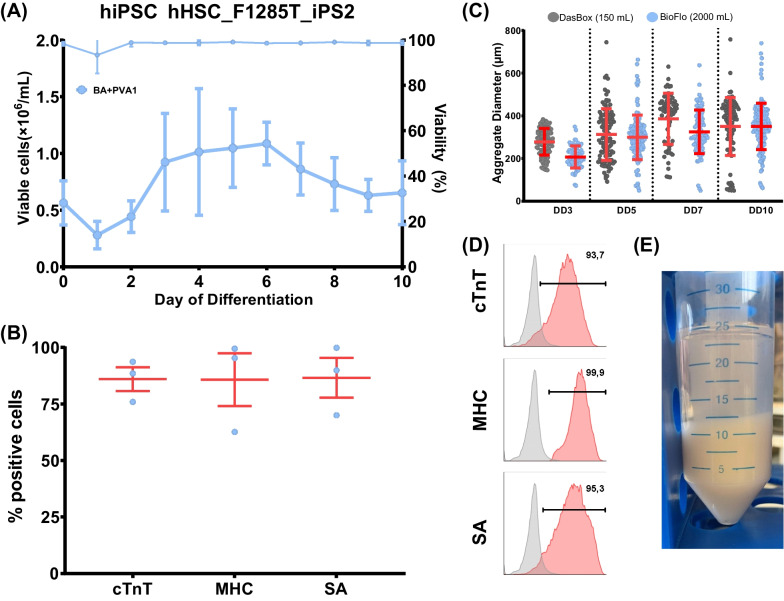
Successful upscaling of differentiation to 2000 mL bioreactor (Bioflo 320). **A** Viable cell number and viability for the hiPSC line hHSC_1285T_iPS2 in a 2000 mL bioreactor differentiated in BA + PVA1 throughout the 10-day differentiation process (n = 3; mean ± s.d.). **B** CM-specific markers cTnT, pan-MHC, and SA for the hiPSC line for differentiation runs in **A** (n = 3; mean ± s.d.). **C** Microscopic analysis of aggregate diameters during the 10-day differentiation process are comparable between an exemplary 150 mL process and a 2000 mL process when utilizing the same cell line hHSC_1285T_iPS2 (each dot represents a single aggregate; mean ± s.d.) **D** Exemplary depiction of the respective markers (cTnT, pan-MHC, SA) for a 2000 mL differentiation process. **E** Total biomass generated in one 2000 mL differentiation process

To further progress the cell number production putatively required for clinical applications, upscaling towards 2000 mL process scale was tested, utilizing another genetically independent hiPSC line (hHSC_F1285T_iPS2 [36]). For cell expansion, hPSCs aggregates were generated at a 500 mL scale (Eppendorf Bioblock) to produce sufficient cells to induce differentiation at a cell density of 1 × 10^9^ hPSCs in 2000 mL scale (Eppendorf Bioflo 320 system). This process enabled the derivation of approximately 1.3 × 10^9^ viable cells/batch (0.65 × 10^6^ ± 0.19 viable cells/mL; Fig. 5A + E) in BA + PVA1 and, importantly, a CM content typically > 85% revealed by flow cytometry on dd10 (Fig. 5B + D). In addition, daily sampling and automatic aggregate size analysis via microscopy revealed that the size pattern of aggregates throughout the 10-day lasting process was highly similar to the established conditions in a 150 mL scale when investigating the same cell line (Fig. 5C), suggesting that the hydrodynamic conditions were comparable in both systems.

## Discussion

This study describes the development and application of novel, chemically defined, and entirely protein-free culture media for the cardiomyogenic differentiation of hPSCs in suspension culture. By omitting protein components, process standardization and reproducibility are promoted, presenting an important step towards the clinical translation of hPSC-CMs beyond the current state of the art [13, 53, 54].

Albumin is the most abundant protein in human blood plasma. It is applied as a common component of cell culture media, as it is known to have a variety of functions, such as the stabilization and protection of media components and cells.

Our data (Fig. 1) suggest that the combined supplementation of RPMI and AA-2P with either PF-68 or PVA1 can take over the roles of albumin as a free radical scavenger and shear protectant.

PF-68 is a foam-reducing and shear-protecting agent in mammalian cell culture [26, 55, 56]. As such, it has been used as an essential component of protein-free media formulations applied to produce recombinant proteins, for example, in baby hamster kidney cells (BHK-21) [57].

The use of a PVA-containing medium has been described for the generation of chimeric antigen receptor T cells (CAR-T cells) [58]. However, the hydrolyzation state and chain length appear to substantially influence respective PVA capacity to promote cell viability and proliferation [43]. It has been demonstrated that the hydrolyzation state mainly affects PVA solubility; a lower state leads to higher water solubility [59]. We have also demonstrated that the biological function of different PVAs to support cardiomyogenic differentiation appears to be influenced by the respective mass and hydrolyzation state (Fig. 1C).

Notably, all chemical components applied in our study are compliant with FDA regulations and are broadly used in the pharmaceutical industry [60, 61]. PVA is known to be biologically inert and is essentially non-toxic in the typically applied concentration range, promoting its frequent use in the cosmetical-, food- and pharmaceutical- industries [61–63]. Thus, although further consultations with regulatory authorities for Advanced Therapy Medicinal Products (ATMP) authorization are required, the BA + PVA1 composition appears straightforward for clinical applications. Similarly, the usage of PF-68 should comply with the regulatory requirements, as no toxicity was observed when the compound was utilized as a drug-carrying agent [64].

It is noteworthy that no concentration adjustment of the applied WNT modulators CHIR and IWP-2 was required in our protein-free approach compared to CDM3, even though recent findings indicate that albumin reduces the activity of CHIR [65]. However, our protein-free differentiations not only yielded comparable cell numbers and CM purities compared to CDM3 (Fig. 2B-E), but the resulting CMs also showed highly equivalent electrophysiological (Fig. 3D-M) and contractile properties (Fig. 3A-C) in line with prior data for CDM3-derived CMs [13].

The resting membrane potential of CMs under protein-free and protein-containing conditions was − 30 to − 40 mV, which is in the range of values described for juvenile CMs [66]. The characteristic, predominantly ventricular-like APs were very comparable in three independent batches of BA + PVA1-derived CMs; some variability in the resting membrane potential, decreasing towards the -90 mV typically found in mature CMs was observed, potentially indicating an ongoing maturation of the differentiated CMs [67].

However, expression levels of structural proteins (e.g., high α-MHC versus low β-MHC levels) confirmed the expected, immature state of both CDM3 and protein-free-derived CMs. Generally, the expression of cytoskeletal proteins and ion channels was in line with previous results (Additional File 1, Figure S4) [51, 68–70].

GO analysis of transcript expression patterns revealed potential differently regulated pathways between different media conditions, such as higher mitotic activity, reduced extracellular structure formation, and reduced TGF-β signaling in BA + PVA1 (Fig. 4C + D).

To our knowledge, the cell yields and CM purities described here (typically 1.5–2 × 10^6^ viable cells/mL at 80–95% CM purity; Fig. 2) are best-in-class and surpass CM yields and purities achieved by other groups in a comparable differentiation approach [47, 71–74]. Similar cell numbers were achieved in the past only by applying genetic enrichment of CMs [15]. Higher CM yields were reported for microcarrier-based differentiation protocols, which, however, require substantial downstream processing [75]. When comparing the results in this paper to our former data utilizing an equivalent differentiation protocol [13], we here produced up to double the viable cell counts utilizing the same hiPSC line (that is, "Phoenix") in both protein-free and CDM3 conditions [13]. A potential reason for these differences may be based on changes in the pre-culture process applied in the current study based on our recently published work [11]. Such changes, which include perfusion-based constant medium exchange after the first 24 h of the process, control of dissolved oxygen, and control of the medium pH, are allowing for an improved expansion and proliferation rate of the hPSCs at the pluripotent state[11]. This may consequently promote higher cell proliferation at early stages of differentiation (days 1–4, Fig. 2B + C) despite maintenance of the general proliferation kinetic patterns in the current study similar to those described previously [13].

Notably, the enabled upscaling of our protein-free process to 2000 mL allows for the production of approximately 1.3 × 10^9^ cells per process run (Fig. 5A). Interestingly, these large-scale processes showed a notable nick in the number of viable cells after 24 h of differentiation (confirmed by reduced viability counts at this stage) compared to growth kinetics observed in 150 mL scale (compare Fig. 2B-E and Additional File 1, Figure S2 to Fig. 5A). The process transition from a 150 mL scale (performed in a DASbox STBR platform) to 2000 mL (performed in a 3000 mL bioreactor controlled by the Bioflo 320 control unit) includes multiple changes, such as those in impeller design and geometry (8-blade impeller to 3-blade impeller) and vessel geometry (flat-bottom to hemispherical bottom). Additional understanding of the potential shear force applied to the aggregates will be required to optimize our process conditions further.

In addition to the above aspects, our protein-free medium formulations should further improve the quality of differentiation media regarding process reproducibility. Many scientists utilizing RHA as a culture medium component have experienced quality fluctuations, especially when utilizing RHA from different vendors and batch-to-batch variations [32]. As RHA is a significant cost driver of the CDM3, utilizing our protein-free formulations allows a price reduction of up to 35% (BA + PVA1 versus CDM3).

Protein-free media formulations also provide new options for process analysis. As shown recently, secreted proteins substantially influence the hPSC-to-CM differentiation [30, 31, 76]. Detailed investigations on the secretome are a potent strategy for advancing the mechanistic understanding of the differentiation process, promoting process robustness, and providing new biomarkers for in-process analysis. However, in protein-containing media, particularly in the presence of albumin, the sensitive assessment of low abundant factors, which may have a dominant influence on lineage differentiation (such as, e.g., antagonists of Nodal signaling such as CER1 and LEFTY1) is challenging [27, 30, 77]. First attempts using simplified protein-reduced media by omitting BSA from the B-27 supplement revealed a substantial improvement in the number of identified secreted proteins along the cardiac differentiation of hPSCs [28]. Our entirely protein-free formulations will further promote the in-depth analysis of the secretome along the entire cardiac differentiation process without the current necessity of protein-depletion steps. Ultimately, by secretome analysis at the differentiation endpoint, CM-secreted factors that impact heart function upon the transplantation of hPSC-derived CMs can be investigated [78–81].

## Conclusions

This work highlights the potential of protein-free media to support the differentiation of pluripotent stem cells into cardiomyocytes in suspension culture, including substantial process upscaling to a 2000 mL scale. The cardiomyocyte yield, purity, and functionality—including contractile and electrophysiological properties—closely match those achieved with protein-containing media, as demonstrated by side-by-side comparisons. Producing 1.5–2 × 10^6 cells/mL at 85–99% underscores the process’ competitive efficiency compared to the state of the art. We anticipate that protein-free differentiation media will also be applicable to other hPSC differentiation approaches, substantially promoting the field and paving the way for robust and cost-effective cell therapy processes. Importantly, the successful process up-scaling to 2000 mL enables the production of approximately 1.3 × 10^9^ cardiomyocytes in a single batch, achieving sufficient cell numbers discussed for replacing cardiomyocyte loss of individual heart failure patients.

### Supplementary Information

Additional file1 (PDF 880 KB)

## Data Availability

The bulk RNAseq data is available via the SRA database via NCBI under accession number prjna1108903. Other data and code shown in this study are available from the corresponding authors upon reasonable request.

## Abbreviations

2D: Two-dimensional
3D: Three-dimensional
AA-2P: Ascorbic acid 2-phosphate
ACTC1: Cardiac muscle alpha actin
AP: Action potential
BCT: Bioartificial cardiac tissue
BHK: Baby hamster kidney
BSA: Bovine serum albumin
CAR T-cells: Chimeric antigen receptor t-cells
Cav1.2: Calcium channel, voltage-dependent, L type, alpha 1C subunit
CDM3: Chemically defined medium, 3 components
CHIR(99021): Selective GSK-3 inhibitor
cTnT/TNNT2: Cardiac troponin T
dd: Day of differentiation
E8: Essential 8 medium
FCS: Fetal calf serum
GFP: Green fluorescent protein
GO: Gene ontology
hESC: Human embryonic stem cell
hiPSC: Human induced pluripotent stem cell
hPSC: Human pluripotent stem cell
hPSC-CMs: Human pluripotent stem cell-derived cardiomyocytes
IWP-2: Inhibitor of WNT production 2
KIR2.1: Cardiac inward rectifier potassium channel
MHC: Pan-Myosin heavy chain
MIXL1: Mix paired-like homeobox
MYLC1v/a: Myosin light chain ventricular/atrial
NAV1.5: Sodium channel protein type 5
NCX1: Sodium-calcium exchanger 1
NKX2.5: Homeobox protein Nkx-2.5
PBS: Phosphate buffered saline
PF-68: Pluronic F-68
PVA: Polyvinyl alcohol
rHSA: Recombinant human serum albumin
RI Y-27632: Rock inhibitor Y-27632
RMP: Resting membrane potential
RPMI (medium): Roswell park memorial insitute medium
RyR2: Ryanodine receptor 2
SA: Sarcomeric actinin
STBR: Stirred-tank bioreactor
TNNI1: Troponin I1
TPM: Tropomyosin
