# Supplementary material for: Protein-free media for cardiac differentiation of hPSCs in 2000 mL suspension culture
Source: Stem Cell Res Ther. 2024 Jul 18;15:213. doi: 10.1186/s13287-024-03826-w (PMC11256493; doi:10.1186/s13287-024-03826-w)
Supplement: Supplementary file 1 — Additional file1 (PDF 880 KB) [file 13287_2024_3826_MOESM1_ESM.pdf]

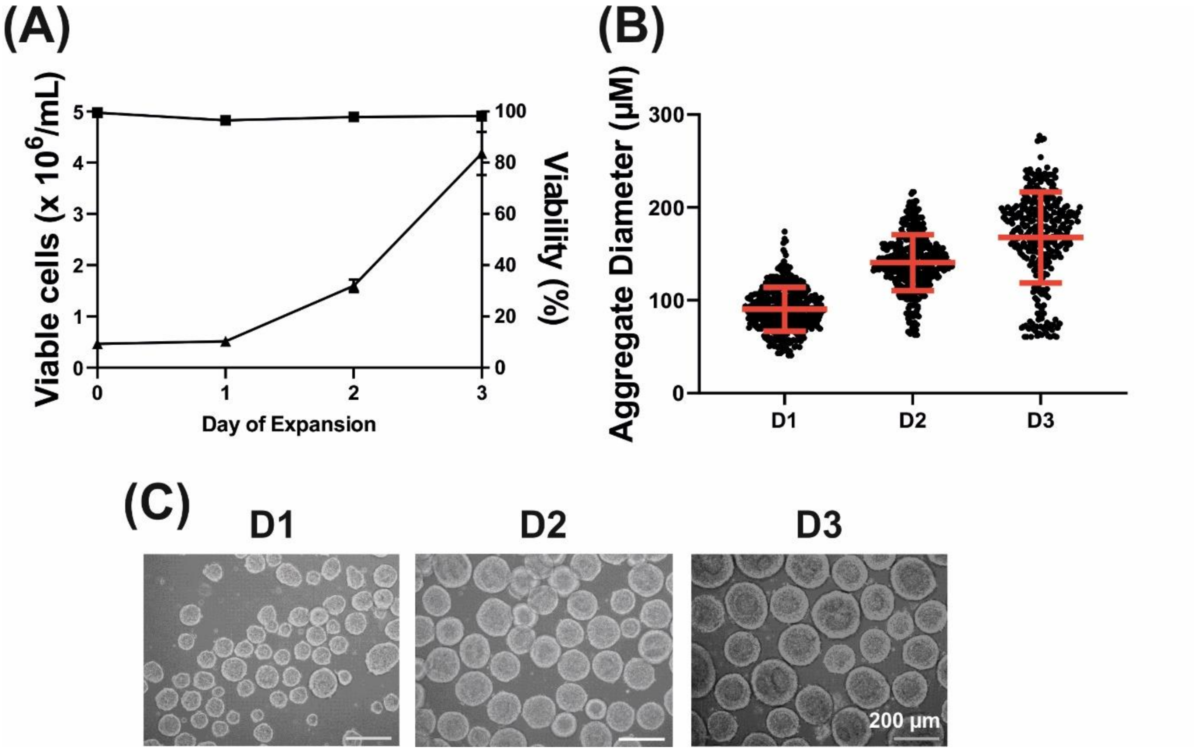

Figure S1: Exemplary pre-culture data during expansion of pluripotent hPSC-aggregates. **A** Viable cell density during the first three days of expansion and respective viability (cell line hESC MIXL-GFP, n = 3, mean ± s.d.). **B** Exemplary aggregate diameter development over the first three days of expansion (every dot represents a single aggregate; red shows mean ± s.d.) **C** Exemplary microscopic pictures of aggregates on pre-culture days 1, 2, and 3 (scale bar = 200 µm).

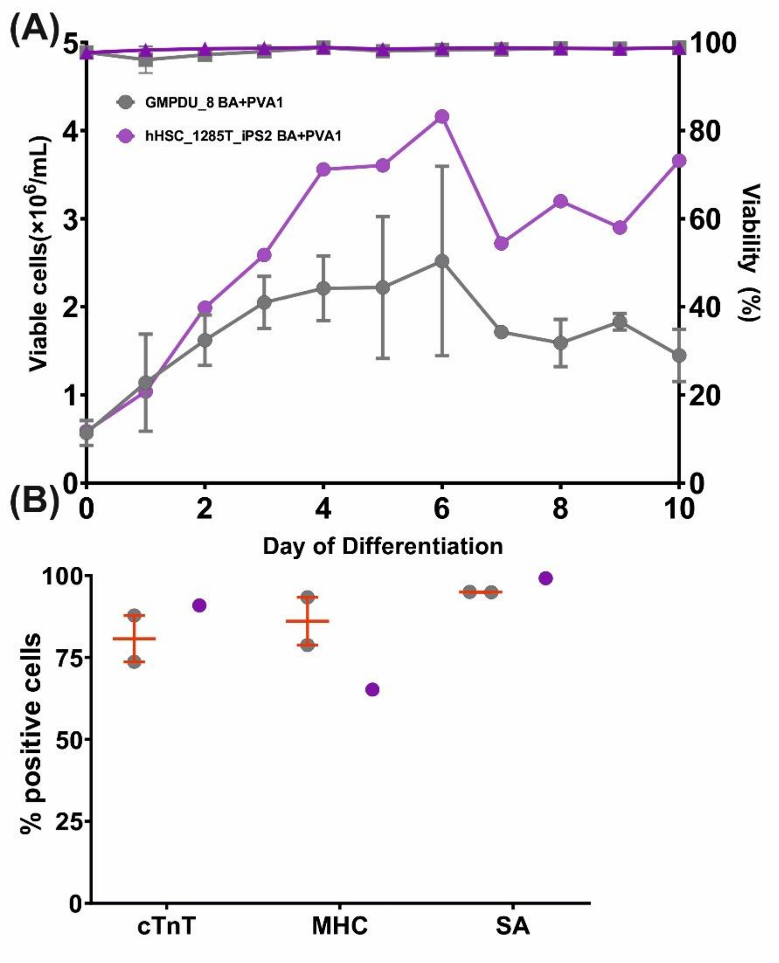

Figure S2: Differentiation kinetics and cardiomyocyte purities for additional cell lines GMPDU and hHSC-1285. **A** Viable cell number and viability for the hiPSC lines GMPDU_8 (grey) and hHSC_1285T_iPS2 (purple) differentiated in BA+PVA1 (GMPDU_8 n = 2 and hHSC_1285T_iPS2 n = 1). **B** CM-specific markers cTnT, pan-MHC, and SA for differentiations depicted in **A** (GMPDU_8 = grey; hHSC_1285T_iPS2 purple).

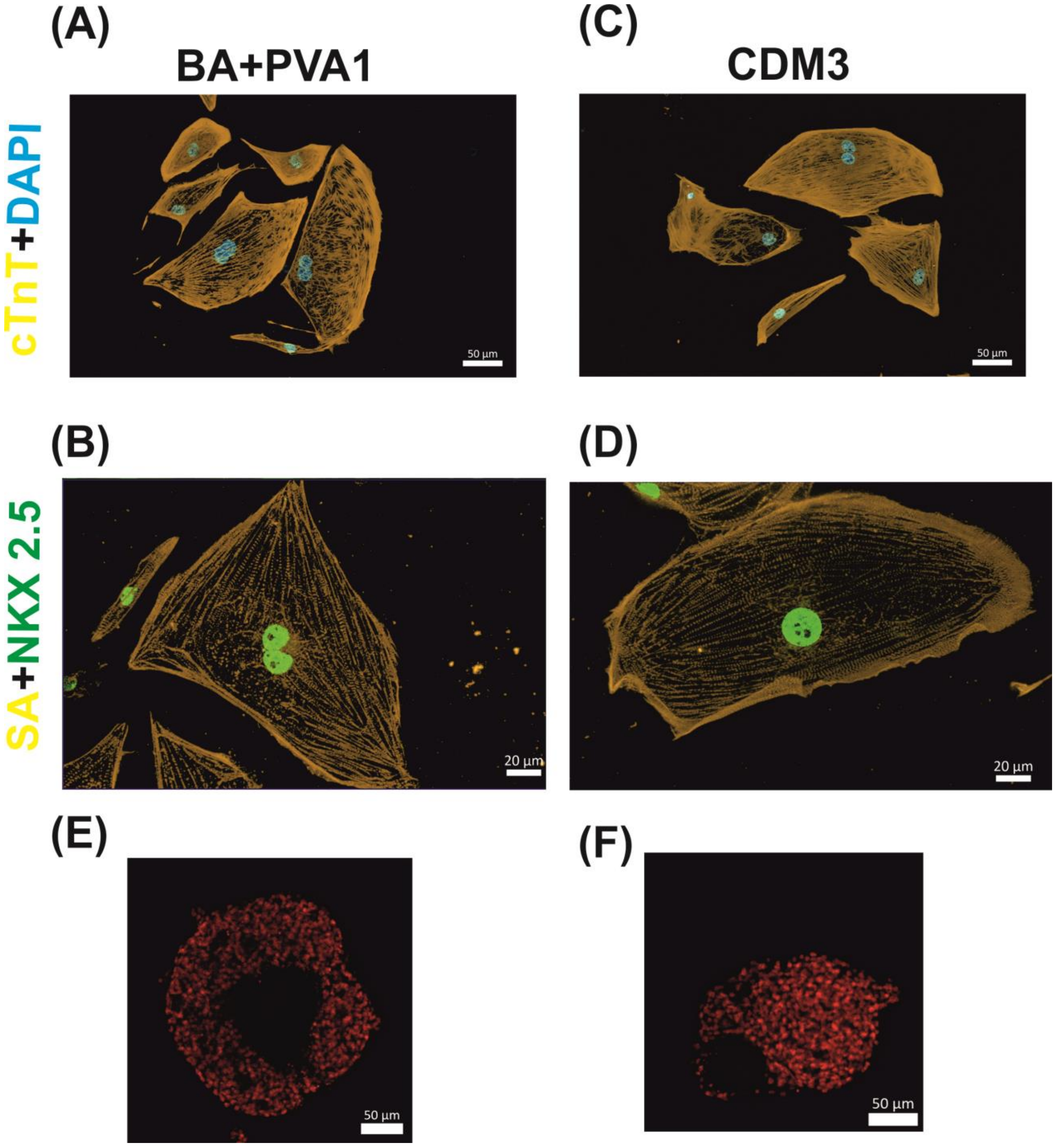

Figure S3: Microscopic analysis of aggregate-derived CMs in BA+PVA1 and CDM3 differentiation conditions. CMs were differentiated as described in BA+PVA1 medium or CDM3, dissociated, and seeded on fibronectin-coated glass slides. After fixation, CMs differentiated in BA+PVA1 were stained for cTnT and with DAPI (**A,** scale bar = 50 µm), or SA and NKX2.5 (**B,** scale bar = 20 µm**)**, or differentiated in CDM3 and stained for cTnT and with DAPI (**C,** scale bar = 50 µm) and SA and NKX2.5 (**D,** scale bar = 20 µm). **E** Aggregates were stained with SytoxDeep

Red, dehydrated, and cleared to reveal typical three-dimensional structures within aggregates after differentiation in BA+PVA1 or (**F**) CDM3 (scale bar = 50 µm).

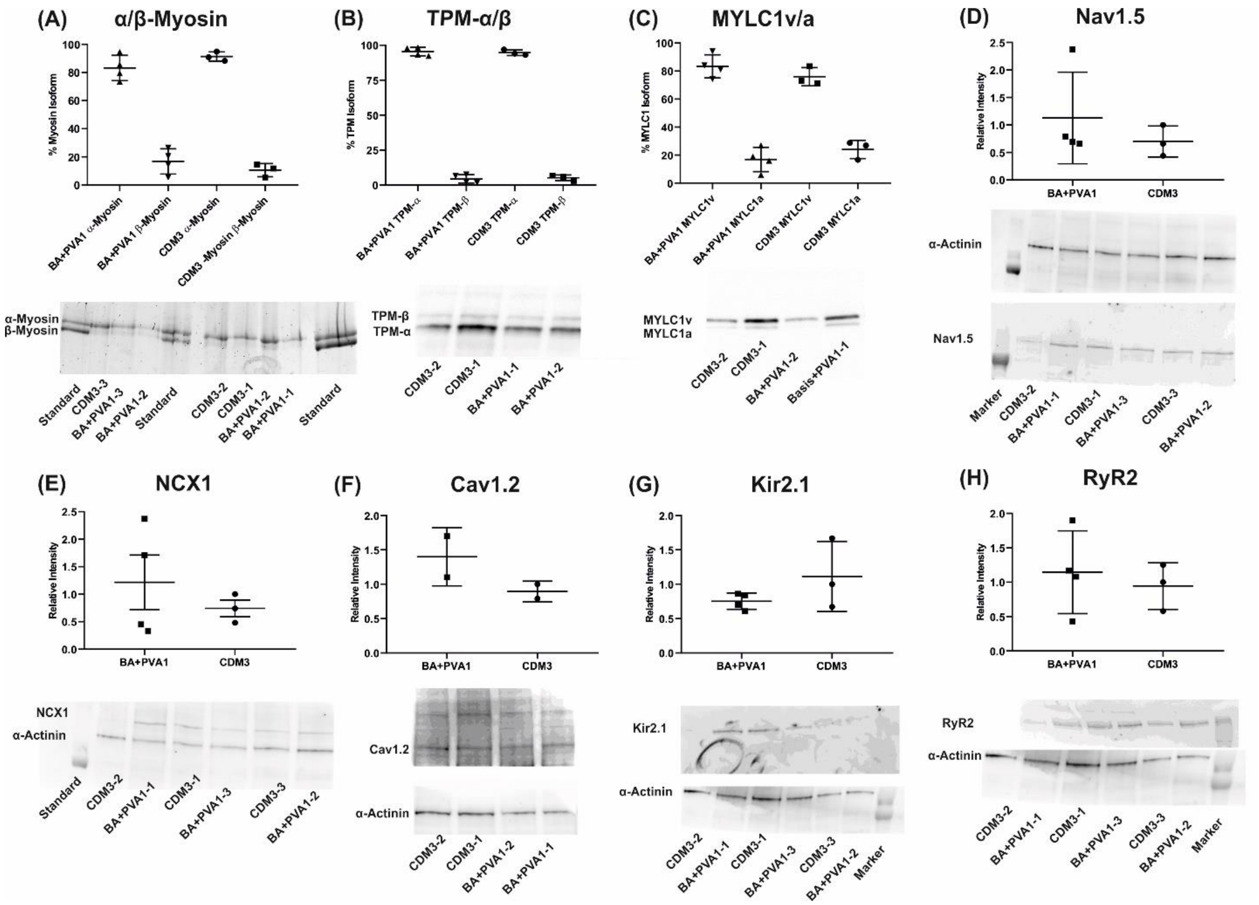

Figure S4: Quantifying cytoskeletal proteins and typical channels in hPSC-CMs. **A** Relative abundance of cytoskeletal proteins in preparations from hPSC-CMs differentiated in CDM3 and BA+PVA1 samples with exemplary SDS-gel separations/western blots (stained with SyproRuby) for α/β-Myosin, B Tropomyosin-α/β, and C MYLC1v/a. Depicted is the relative intensity of typical channels in preparations from hPSC-CMs differentiated in CDM3 and BA+PVA1 samples and exemplary western blots. **D-H** Relative intensity for typical cardiac channels. All relative intensities were normalized to α-Actinin for the amount of protein applied and signal intensity compared to one CDM3 sample to allow cross-gel comparison. Channels analyzed were: **D** Nav1.5, **E** NCX1, **F** Cav1.2, **G** Kir2.1, and **H** RyR2. For every sample, at least two gel runs were performed, and the average value is depicted (n = 3/4 for CDM3/BA+PVA1) except for Kir2.1 n = 2/2). Shown are single values and mean ± s.d.

Table S1: Antibodies used for cardiomyocyte purity assessment

**Antigene**	**Derived from**	**Vendor; Cat.No.**	**Dilution**
cTnT	Mouse	Invitrogen; MA5-12960	1:200
SA	Mouse	Sigma-Aldrich; A7732	1:800
Pan-MHC (MF-20)	Mouse	Hybridoma Bank	1:20

Table S2: Antibodies used for western blot analysis

**Antigene**	**Derived from**	**Vendor; Cat.No.**	**Dilution**
Tropomyosin-α/β	Mouse	Sigma-Aldrich; T2780	1:1000
MyLC1 v/a	Rabbit	Thermo-Fisher; PAS-49205	1:1000
Cav1.2	Goat	Abcam; ab81980	1:300
NaV1.5	Rabbit	Almonde; ASC-005	1:200
Kir2.1	Rabbit	Abcam; ab10975	1:200
NCX1	Rabbit	Abcam; ab177952	1:1000
RYR2	Mouse	Thermo-Fisher; MA3-916	1:500
Alpha-Actinin (Control)	Mouse	Sigma-Aldrich; A7811	1:1000
Secondary antibody anti-mouse	Goat	BioRad; 172-1011	1:3000
Secondary antibody anti-rabbit	Goat	BioRad; 170-6515	1:3000
Secondary antibody anti-goat	Rabbit	BioRad; 172-1034	1:3000

Table S3: Strongest regulated transcripts according to RNA-seq analysis

**Gene Name**	**-$\log_{10}$ p-value**	**Log_2 Fold-change BA+PVA-CDM3**

ADAM11	1.79	58.02479
PDE3A	1.55	15.59112
CCND2	1.54	33.14855
ADRA2B	1.54	26.24646
TRH	1.45	50.90951
SYTL4	1.41	13.48804
CDCA7L	1.38	18.1518
BIRC5	1.37	29.77719
IGFBP5	1.35	21.28509
KIAA1614	1.35	8.339952
GCK	1.34	13.04379
RASL12	1.34	12.3104
CCND1	1.31	21.07987
LGALS3	1.31	7.5258
TECRL	1.28	9.799983
ADA2	1.25	8.784085
PHACTR3	1.24	7.961165
DHCR24	1.23	14.91588
GJA5	1.21	6.276046
ECHDC2	1.2	19.82894
RRM2	1.2	18.82115
AL159166.1	1.19	6.852435
MKI67	1.19	26.38959
AURKB	1.18	11.6574
PPP1R14C	1.17	30.49163
FGD4	1.16	7.803068
TROAP	1.16	9.026348
C10orf82	1.15	6.396747
PLXNA2	1.15	29.71167
KIF20A	1.14	20.04878
PIMREG	1.13	8.802384
FOXM1	1.12	11.02267
MMP23B	1.12	8.705835
RXRG	1.11	27.23845
ZFHX4	1.09	6.889713
ABO	1.08	5.219991
CCNA2	1.08	21.94887
GYPC	1.08	18.68681
JHY	1.08	5.457327
GTSE1	1.08	9.613106
MARCKS	1.07	6.805234
NUSAP1	1.07	17.58693
CTHRC1	1.07	9.321156
CDC20	1.07	13.26178
DEPDC1	1.06	13.28595
IRX4	1.06	11.62577
TBCE	1.06	6.422977
GADL1	1.05	4.702897
ASPG	1.05	5.477021
ADPRHL1	1.05	17.29029

COL9A1	1.04	16.95482
PRRG3	1.04	9.192835
KIF4A	1.02	8.494659
KIF2C	1.02	12.90728
CDCA8	1.02	8.678613
TPX2	1.02	23.63141
APOLD1	1.01	5.681424
CRIP1	1.01	8.105311
MYBL2	1.01	9.477924
CCNB2	1	10.26296
ADAMTS2	-1	15.13353
MMP2	-1	15.84909
SEZ6L2	-1.01	9.262579
PDLIM3	-1.01	4.447175
TGFBI	-1.02	4.578159
PRKG1	-1.03	15.91347
MKX	-1.04	6.957228
FBXO2	-1.04	5.716643
PCDH9	-1.04	9.751363
RUNDC3A-AS1	-1.04	4.971169
NDNF	-1.04	12.45221
SLC6A15	-1.05	6.275683
LMO2	-1.05	10.33906
TAGLN	-1.05	47.22251
GBP2	-1.06	4.797576
HLA-B	-1.06	5.984086
ODAM	-1.07	8.764513
KIF5C	-1.07	20.96058
CEACAM21	-1.08	4.881626
B3GAT2	-1.08	5.347077
NPW	-1.08	5.039213
SLC15A2	-1.09	5.17761
SLC16A3	-1.1	6.290675
HSPB6	-1.1	13.10599
PEG10	-1.11	99.35135
HOPX	-1.11	5.204495
CRMP1	-1.11	21.19561
MMP24	-1.14	11.46256
TMEM132B	-1.16	7.951642
TESC	-1.16	11.58072
GRID1	-1.16	12.29856
ELF4	-1.16	5.786731
SPSB1	-1.18	7.85112
ARG2	-1.18	9.278507
OLFM2	-1.18	7.312496
SLC24A3	-1.19	15.62852
ANKRD1	-1.2	11.57968
HAPLN1	-1.2	21.07987
GCNT1	-1.22	6.381534
EGLN3	-1.23	7.555221

HPX	-1.24	7.826332
ITGA2	-1.24	10.26296
CLU	-1.25	38.2376
DLK1	-1.25	9.561064
SSC5D	-1.26	7.793913
NPY	-1.27	8.435061
FST	-1.27	7.023554
RYR3	-1.28	9.327787
HCG22	-1.28	7.060182
TRIB1	-1.29	20.03916
AQP10	-1.29	16.7429
EHD2	-1.31	11.48397
MXRA5	-1.32	7.606334
FSTL5	-1.34	12.44754
NR4A3	-1.34	7.794994
NR4A1	-1.35	10.19306
INHA	-1.39	13.53879
CEMIP	-1.39	8.567059
SLC35F1	-1.4	11.79517
ADAMTSL2	-1.4	15.55994
SHISA2	-1.4	16.09766
SGCD	-1.42	9.262579
COL4A4	-1.45	9.751363
LDLRAD4	-1.46	27.53353
AMER3	-1.46	13.33193
KCNK3	-1.46	15.18494
HSPA6	-1.48	10.07811
JUNB	-1.5	15.74483
ACTA1	-1.51	12.28808
CCDC80	-1.52	34.21758
PMEPA1	-1.53	21.65309
THBS1	-1.54	11.52178
SYNPO	-1.58	11.05389
PCDH1	-1.63	13.26636
GJD2	-1.64	12.0475
POU2F2	-1.65	12.14822
NRP2	-1.67	59.4127
GABRP	-1.67	12.83955
FOSB	-1.68	14.79961
CSDC2	-1.78	14.86922
MECOM	-1.87	26.3475
CRISPLD2	-1.87	50.64652
ANXA1	-1.96	18.30897
ITPKB	-2	38.9598
CNTN5	-2.18	119.9962
CFC1	-2.24	56.57412
SV2C	-2.86	83.54353
